# A novel approach to pulmonary bronchial tree model construction and performance index study

**DOI:** 10.3389/fphar.2023.1254804

**Published:** 2023-11-23

**Authors:** Yang Liu, Weiyan Qiu, Longyu Li, Rongchang Chen, Yan Kang, Shuangchen Ruan

**Affiliations:** ^1^ College of Applied Sciences, Shenzhen University, Shenzhen, China; ^2^ Medical Health and Intelligent Simulation Laboratory, Health Science and Environmental Engineering School, Shenzhen Technology University, Shenzhen, China; ^3^ State Key Laboratory of Respiratory Disease, National Clinical Research Center for Respiratory Disease, The National Center for Respiratory Medicine, Guangzhou Institute of Respiratory Health, The First Affiliated Hospital of Guangzhou Medical College, Guangzhou, China

**Keywords:** model organisms, respiratory disease, pulmonary disease, bronchial tree, morphology model

## Abstract

The demand for respiratory disease and dynamic breathing studies has continuously driven researchers to update the pulmonary bronchial tree’s morphology model. This study aims to construct a bronchial tree morphology model efficiently and effectively with practical algorithms. We built a performance index system using failure branch rate, volume ratio, and coefficient of variation of terminal volumes to evaluate the model performance. We optimized the parameter settings and found the best options to build the morphology model, and we constructed a 14th-generation bronchial tree model with a decent performance index. The dimensions of our model closely matched published data from anatomic *in vitro* measurements. The proposed model is adjustable and computable and will be used in future dynamic breathing simulations and respiratory disease studies.

## 1 Introduction

Chronic obstructive pulmonary disease (COPD) is a common disease with pulmonary airflow restriction. It is currently the third leading cause of death globally, mainly reflected in the destruction of lung structures or morphological changes. The advent of computed tomography (CT) has revolutionized radiology and gradually altered the gold standard in diagnosing and treating COPD. Radiology is now preferred when COPD is suspected. Although the GOLD report diagnostic criterion is still a lung function test, it also mentions that approximately 30% of COPD patients have visible bronchiectasis on CT, which is associated with an increased frequency of exacerbations and mortality (Martinez-Garcia et al., 2021). The heterogeneity of COPD also shows that personalized lung tissue morphology has played a significant role in the occurrence and development of the disease.

High-resolution CT examination of the lung is used to diagnose and monitor disease of the lung tissue and the airways. Because the bronchus of the lungs has approximately 23 generations, the diameter decreases progressively generation by generation. Some bronchi are invisible in CT because of its limited resolution. Understanding the tracheal tree’s morphology facilitates the study of different levels of bronchiectasis.

It has been over 50 years since Weibel built the earliest lung model, the Weibel A model, through his research on pulmonary morphology (Weibel et al., 1963). It assumes that an airway consists of cylindrical geometries, and each bronchus symmetrically branches into two bronchial tubings. Researchers have recently developed models that are more similar to the real bronchial tree morphologically and functionally. Huang et al. (2020) segregated the bronchial tree models in TB and alveolar models into three categories. The first category is models developed from the Weibel A model. They are symmetrical bifurcated bronchial structures with cylinders of various lengths and diameters, and most of the models have *in vitro* results available for comparison. In 2017, Ciciliani et al. (2017) constructed the bronchial tree geometry using SolidWorks. Based on Finlay’s findings, the alveolar space is not present. They later used ANSYS to perform the CFD simulation and obtain the particle deposition results. In 2018, Koullapis et al. (2018) added the alveolar sacs model to Weibel’s bifurcation modeling and performed the computational fluid-particle dynamics simulation. These morphology geometries always require excessive simplifications while losing information about the original bronchial tree structure. The second category is the realistic models generated from high-resolution CT or MRI imaging. Islam et al. (2017) built a 17-generation human respiratory tract model from the high-resolution CT image from Schmidt et al. (2004). Other researchers have also created realistic models. They are not widely used as respiratory tract geometry is always different from individual to individual (Rajaraman et al., 2020; Mohite et al., 2020; Farghadan et al.,2020; Srivastav et al.,2019; Poorbahrami and Oakes, 2019). The third category is computer-generated models. These models are generated using computer algorithms based on the data from realistic models. They are more accurate than the Weibel model and less computing demanding than the CT-based models. Researchers can generate models of different conditions for parametric study. These algorithms came from a two-dimensional tree generation Monte Carlo method. Tawhai et al. (2000) extended the two-dimensional bifurcation algorithm to a three-dimensional tree-growing algorithm to generate a lung model. Kitaoka, 2011 and Kolanjiyil and Kleinstreuer, 2016 developed a four-dimensional model (a three-dimensional model varies with time) capable of generating airway pathways and alveolar structures with realistic characteristics of bronchial airways. He established nine basic laws and four supplement laws, which are difficult for other researchers to replicate. This paper studied several bifurcation algorithms and found that the space-dividing algorithm performs the best. Then, we originated our bronchial tree model, based on the idea that the bronchial tree is supposed to transport air to the entire lung space in the best and easiest way. We then used three performance indices to evaluate the modeling and find the best-fit parameters.

## 2 Approaches

### 2.1 Medical data and schematic flowchart

Our dataset comes from open longitudinal research from Guangzhou’s first medical school in Guangzhou, China, from 2010 onwards. Among the 400 individuals who attended this research, we selected one male patient of average height and weight among all the attendants. Chest multi-slice spiral computed tomography (MSCT) was performed with a multiple-layer scanner with a 1 mm calibrated volume under 100 kV and a 40 mA current. The spinning time was 0.75 s with a spacing of 1.2 mm. The FOV was 40 cm. Scanning was performed from toe to head to eliminate the artifacts. CT images were reconstructed with the software, which provided a thickness of 1 mm. The total radiational dose was 1–3 mSv. Patients were lying on the back, holding their breath after deep breathing, and the scanning did not need to inject the contrast agent.

**FIGURE 1:**
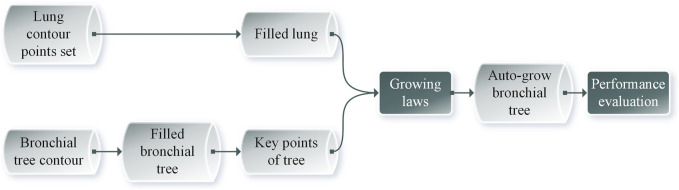
Schematic flowchart of this study.

The schematic flowchart is shown in Figure 1. First, 561 Dicom images were imported in Mimics 19^®^. Then, the bronchial tree three-dimensional models were extracted from the CT images, with medium weakness and region grow. Then, we exported the.txt file and extracted the centerline. The left lung three-dimensional object was exported as 16,165 outline points, and 16,453 outline points represented the right lung. Then, we filled the space between the boundary points with a 1 mm interval in x, y, and z directions. The resulting 3,708,588 points filled the left lung and 3,968,949 points filled the right lung. The number difference was because, for most humans, the right lung has a larger size than the left one. Then, we set up a sampling constant to downsample this points cloud, which reduced the size of the matrix. The selection of the sampling constant needs to be analyzed for each situation. The root point and the three starting points (near, side, and father) are then located to initialize the bronchial branch. Three different automatic growing methods will be explained in detail in the following sections. These methods were used to grow the airway tree automatically. Then, we used three performance indices to evaluate the bronchial tree and the parameter setting to find the model with the best performance.

### 2.2 Branching laws based on pre-set data

Based on the bronchial tree basic algorithm, our first three-dimensional model of the human airway tree was generated from four starting points: a pre-set branching diameter, branching angles, turning angles, and a lung outline. The main idea of this algorithm is as follows: The bronchial tree is supposed to follow a similar pattern before and after, men and women. The diameters and branching angles should match the recorded data measured ex vivo published by other researchers. The pre-set data algorithms were composed of three fundamental laws and two space separation laws.

Branching law - pre-set data algorithms

Law 1: Branching is dichotomous.

Law 2: The parent branch and its two daughter branches lie in the same plane, called the branching plane.

Law 3: The angle between a parent branch and its daughter branch is this daughter’s branching angle.

Law 4: The region supplied by a parent branch is divided into two daughter regions by the “space-dividing plane.” The angle between the space-dividing and branching planes is the turning angle.

Law 5: Overflow (branch outside the lung outline) terminate the branch.

First, we needed to define four starting points, the beginning point and the father, near, and side points, to initialize the three starting branches. The pre-set branching and turning angles determine where the new branch grows to, from the near point and the branching plane. With this method, the bronchial tree’s shape largely depends on the pre-setting data. The diameter we used in this study came from the average branch length measured by Yeh and Schum (1980), and we performed a curve fitting. The branching angles were supposed to be 60°to 65°, and the turning angles were controlled near 90° . These two angles were used to solve the space-dividing plane and then solve the quadric equations in six unknowns for eight groups of possible grow points. Two closer solutions were selected at first. If the newly developed line was close to existing bronchi or went across the lung outline, the solution was changed to another one until the eight possible grow points were used up. If no solutions are selected, then the pre-set branching angle will be reduced with a random percent off to solve other possible grow points. After one side is done, the near seed and the side seed switch positions to find the other two possible grow points. Except for these three fundamental laws, the bronchial tree is auto-growing based on the pre-obtained bronchial tree data and the space restriction. The pre-obtained data come from our semi-auto measurements using 3-D Slicer^®^ and the published data were measured by previous researchers. The data included the length, width, branching angle, and rotation angles of each level of the bronchial tree. The data we used for the growth are shown in Table 1.

**TABLE 1:** Initial parameters set up for pre-set data algorithms.

Bronchi length L (mm)	(17.8 9.65 9.95 10.1 8.9 9.62 8.67 6.67 5.56 4.46 3.59)
Major branching angle (deg.)	(65 65 70 65 70 65 65 65 65 65 65)
Minor branching angle (deg.)	(65 65 70 65 60 65 60 65 65 65 65)
Major turning angle (deg.)	(33 34 22 20 18 19 22 28 22 33 34)
Minor turning angle (deg.)	(33 34 22 23 25 24 25 31 25 33 34)

### 2.3 Branching laws based on flow rate division

#### 2.3.1 Relationship between the flow rate and diameter

Researchers have suggested an optimum relationship between the flow rate and diameter in a circular rigid tube with a constant diameter. Murray, (1926) first proposed the relationship between the diameter and the flow rate, and other researchers later modified them.

$$Q=Cd^{n},$$ (1)

where d is the diameter, Q is the flow rate, n is the diameter exponent, and C is a constant depending on the organ and the fluid.

Considering the conservation of the mass before and after the branching, we can relate the diameter of the parent $d_{0}$ to the diameters of the daughters $d_{1}$ and $d_{2}$ , so we have

$$d_{0}^{n}=d_{1}^{n}+d_{2}^{n}.$$ (2)

After defining the flow-dividing ratio r as the ratio of the smaller daughter branch,

$$d_{1}=d_{0}r^{1/n}$$ (3)

$$d_{2}=d_{0}{\left(1-r\right)}^{1/n}.$$ (4)

Based on a minimum energy loss principle, the value n is suggested to be 3. The theoretical value of this morphometric value for the airway is still unclear in the academic community. Here, we use the value 3 to solve the angles.

$$\cos\theta_{1}=\left[1+r^{\frac{4}{n}}-{\left(1-r\right)}^{\frac{4}{n}}\right]/2r^{2/n}$$ (5)

$$\cos\theta_{2}=\left[1+{\left(1-r\right)}^{\frac{4}{n}}-r^{\frac{4}{n}}\right]/2{\left(1-r\right)}^{2/n}.$$ (6)

#### 2.3.2 Branching laws based on the flow rate algorithms

The second three-dimensional model of the human bronchial tree was generated from three initial points and a lung outline based on the bronchial tree flow rate algorithm. The chief idea of this algorithm is as follows: the volume flow rate in the bronchial tree transport system is supposed to be conservative. Each bifurcation separates the flow rate to go to the farthest end in the best and most accessible way. For each furthermost branch, the flow rate is supposed to support its covered space. The flow rate algorithms are composed of seven laws.

Branching law–flow rate algorithms

Law 1: Branching is dichotomous.

Law 2: The parent branch and its two daughter branches lie in the same plane, called the branching plane.

Law 3: A space-dividing plane divides the region supplied by a parent branch into two daughter regions. The space-dividing plane is perpendicular to the branching plane, and it reaches out to the parent branch.

Law 4: The volumetric flow rate is conserved after branching, as shown in Eq. 2.

Law 5: The flow-dividing ratio r is set to be the volume-dividing ratio.

Law 6: Diameters and branching angles of the two daughter branches are determined by Eqs 3–6, respectively.

Law 7: Empty data set and overflow (branch outside the lung outline) terminate the branch.

### 2.4 Branching laws based on the space-dividing algorithms

Our space-dividing algorithm generated the third three-dimensional model of the human airway tree from three starting points and the lung outline. The core idea of this algorithm is as follows: The bronchial tree is supposed to transport air to the entire space of the lung using the best and easiest way. If there is a branch, it is considered to transport air toward the center of the demarcated space. The basic laws are composed of three basic laws and space separation laws.

Branching law–space-dividing algorithms

Law 1: Branching is dichotomous.

Law 2: The parent branch and its two daughter branches lie in the same plane, called the branching plane.

Law 3: the branching plane cuts the supporting area into upper and lower sections.

Law 4: The center of the divided space is where the newer branch grows toward, with a constant ratio a . If the length of the growing branch exceeds the limit, we reduce with a constant length ratio b . If the dividing angle is obtuse, we use a correction ratio c to reduce this angle.

Law 5: The empty data set and overflow (branch outside the lung outline) terminate the branch.

### 2.5 Modeling performance index

Failure branch rate, volume ratio, and coefficient of variation of terminal volumes were used to quantify the bronchial tree model’s ability to transport air evenly to all the space in the lung region.

#### 2.5.1 Failure branch rate ( $R_{fb}$ )

The branches extending beyond their quantified region are terminated, violating the concept that fluid should be delivered homogeneously in the space available for the tree. The available space is correspondingly eliminated from the consideration of the space to grow branches. We call these branches “failure terminal branches,” and $R_{fb}$ is calculated as the ratio of the cutoff branches divided by the total branch numbers. The smaller $R_{fb}$ represents a better branching quality.

#### 2.5.2 Volume ratio ( $V_{r}$ )

The space of the bronchial tree structure (the part within the lung lobes) is divided by the volume of both the lung lobes. A tree with a smaller $V_{r}$ representing it leaves a larger space inside the lung lobes, which can be filled with alveolars to perform the blood-oxygen exchange. A smaller $V_{r}$ represents a better design.

#### 2.5.3 Coefficient of variation of terminal volumes ( $C_{v}$ )

We can grow multiple generations of the bronchial tree and set the parted spaces in the last loop as the terminal regions supplied by each corresponding terminal branch. $C_{v}$ is defined as the coefficient of variation of the volumes of those terminal regions. The value of $C_{v}$ should be small for uniform space division.

## 3 Results

### 3.1 The bronchial tree growth

**FIGURE 2:**
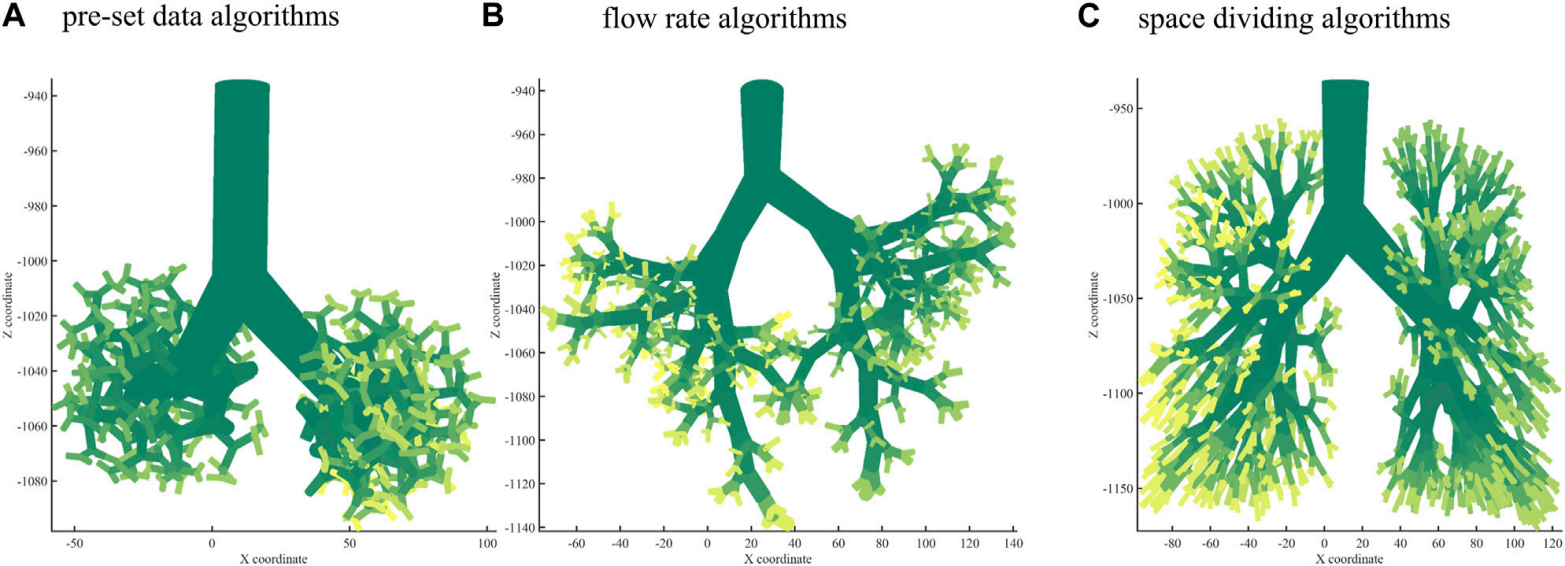
Bronchial tree with three algorithms. **(A)** A 10th-generation bronchial tree model created with pre-set data algorithms. **(B)** A 10th-generation bronchial tree model created with flow rate algorithms. **(C)** A 10th-generation bronchial tree model created with space-dividing algorithms.

Using the branching laws based on the pre-set data algorithm, a bronchial tree was generated to the 10th generation, as shown in Figure 2A. Using the branching laws with the flow rate algorithm, the bronchial tree was generated to the 10th generation, as shown in Figure 2B. Figure 2C shows the bronchial tree structure using the branching laws based on the space-dividing algorithm. The bronchial trees were grown to the 10th generation for all three methods. The space-filling was relatively poor for the first two algorithms, especially in the upper part of the lung. In Table 2, the volume rate and $C_{v}$ also show this difference. We tried to alter the parameters used in the first two laws; however, as the branching angle, length, and diameter vary from branch to branch, left to right, and top to bottom, it was very difficult to define a suit of parameters to grow a perfect bronchial tree model. Extra corrections will be needed to make the branches fill the lung shape better. The third law was based on space-filling function and fills the given space perfectly. In addition, the first two algorithms use the lung outline to confine the branches, resulting in a high failure branch rate (also shown in Table 2). This will largely affect our CFD simulation in future studies. Furthermore, only the third law allows for obtuse angles, which actually exist in the real bronchial tree. Table 2 shows the performance indices for the three branching laws. The pre-set data and flow rate algorithms separated space with a higher $C_{v}$ , and space-dividing algorithms separated the space well, leaving an acceptable failure branch rate. Pre-set data and flow rate algorithms had a smaller volume ratio; this is because they did not occupy all the space in the lung, and therefore, they are not suitable for future studies.

**TABLE 2:** Three branching algorithms and the performance indices.

	$R_{fb}$	$V_{r}$	$C_{v}$
Pre-set data algorithms	8.7%	30.85%	0.361
Flow rate algorithms	2.5%	25.53%	0.387
Space-dividing algorithms	3.1%	32.59%	0.192

### 3.2 Study on the coefficients setting

#### 3.2.1 The sampling constant

First, we filled the left and right lung contours as point clouds and used a sparse constant to fasten the calculation speed. If the sparse constant is too small, the calculation will take much longer and will not bring extra information; if the sparse constant is too large, it will affect the precision of the analysis. We performed the test tree growing of 10 generations with the sampling constants 10, 300, 600, and 9,000. The failure branch rate was 2.83%, 2.54%, 2.83%, and 25.4%, respectively. Then, we chose a sampling constant of 300 to perform the bronchial tree growth of 10 generations.

#### 3.2.2 The growth ratio **a**

The constant growth ratio a was used when we calculated the length of a new branch. After we cut the branching region into two portions, the new branch should go toward each portion’s center. It should not grow exactly to the center of each part as this is not an effective way to transport air to both parts, and fluid will not prefer to make sharp turns. Therefore, the growth ratio *a* was defined to multiply the vector from the new father point to the center of each of the two portions as the length of the new bronchus. From the published data and our observations, ratio a should be less than 0.5 but greater than 0.3. To find the best performance, we used a = 0.35, 0.40, and 0.45 to grow the bronchial tree and compare the three performance indices, as shown in Table 3 below.

**TABLE 3:** Study of growth ratio a with performance indices.

	$R_{fb}$	$V_{r}$	$C_{v}$
a = 0.35	2.9%	31.39%	0.193
a = 0.40	3.1%	32.59%	0.192
a = 0.45	3.7%	33.59%	0.194

$R_{fb}$ and $C_{v}$ showed that a = 0.40 performed the best in transporting the air more evenly, but a = 0.35 had fewer failure branches. This means a = 0.35 creates more successful branches, leaves less empty volume, and separates the space acceptably evenly. $V_{r}$ is the volume ratio of the bronchial tree over the whole lung, and it is supposed to be smaller when the growth ratio a is smaller. We can say that a = 0.35 performs the best.

#### 3.2.3 The length limit and the length ratio **b**

When using this method to grow the bronchial tree, sometimes we inevitably cut a long and thin region to grow, and the branch may be too long; therefore, we have to use a length limit to correct the length. As shown in the previously measured data by Yeh and Schum (1980), the bronchus length should not exceed 1.5 times the measured length. All the lengths should not exceed 20 mm; the second generation should not exceed 8.72 mm, and 3.56, 1.93, 1.99, 2.02, 1.78, and 1.92 mm in turn. The length ratio b is another ratio multiplied by growth ratio a while the length of the new branch exceeds the limit, and we tested four values: b = 0.3, 0.4, 0.5, and 0.6. The performance index is shown in Table 4 below.

**TABLE 4:** Study of length ratio b with performance indices.

	$R_{fb}$	$V_{r}$	$C_{v}$
b = 0.3	4.9%	30.61%	0.194
b = 0.4	3.5%	31.78%	0.199
b = 0.5	3.1%	32.59%	0.192
b = 0.6	4.7%	33.43%	0.188

It can be seen that b = 0.5 gave the best failure branch rate and with a decent volume ratio. For b = 0.3, 0.4, and 0.5, $C_{v}$ did not vary too much. So overall, b = 0.5 gave better performance and will be used in the later study.

#### 3.2.4 The angle correction coefficient c

We observed the human lung and obtained opinions from medical doctors. Some angles inside the human lung may be obtuse but the proportion is limited. Once the calculated angle was obtuse, we added a correction to the modeling and made this angle slightly smaller, and if it was still obtuse, we kept it as it is. The constant c is a fraction. If the angle between the father bronchus vector and the daughter bronchus vector is obtuse, we multiply c to that angle.

Compared with the other three ratios, c=0.3 had a relatively better performance, with a slightly smaller $R_{fb}$ and a slightly better $C_{v}$ , as shown in Table 5. Additionally, c=0.3 will be used in the later modeling.

**TABLE 5:** Study of proportional ratio c with performance indexes.

	$R_{fb}$	$V_{r}$	$C_{v}$
c = 0.2	3.3%	32.58%	0.190
c = 0.3	3.1%	32.59%	0.192
c = 0.4	3.1%	32.60%	0.198
c = 0.5	3.1%	32.62%	0.202

### 3.3 Best performing bronchial tree modeling

**FIGURE 3:**
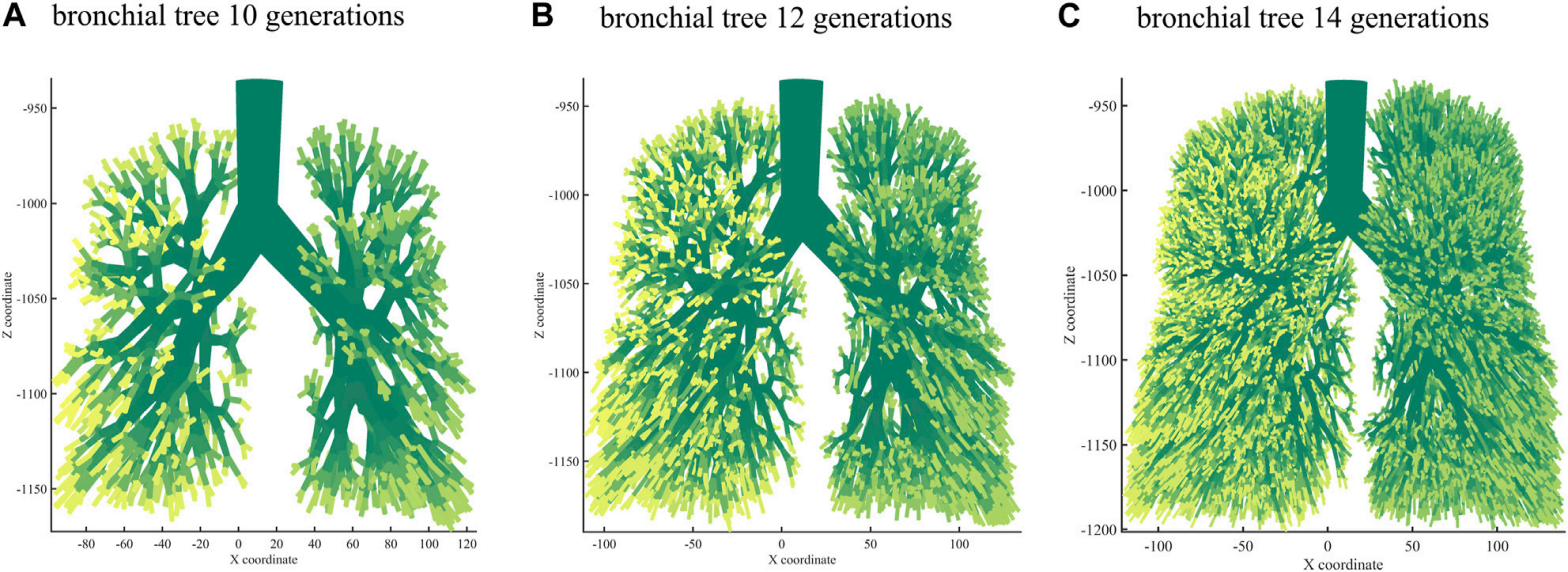
Bronchial tree model of the 10th **(A)**, 12th **(B)** and 14th **(C)** generations.

**FIGURE 4:**
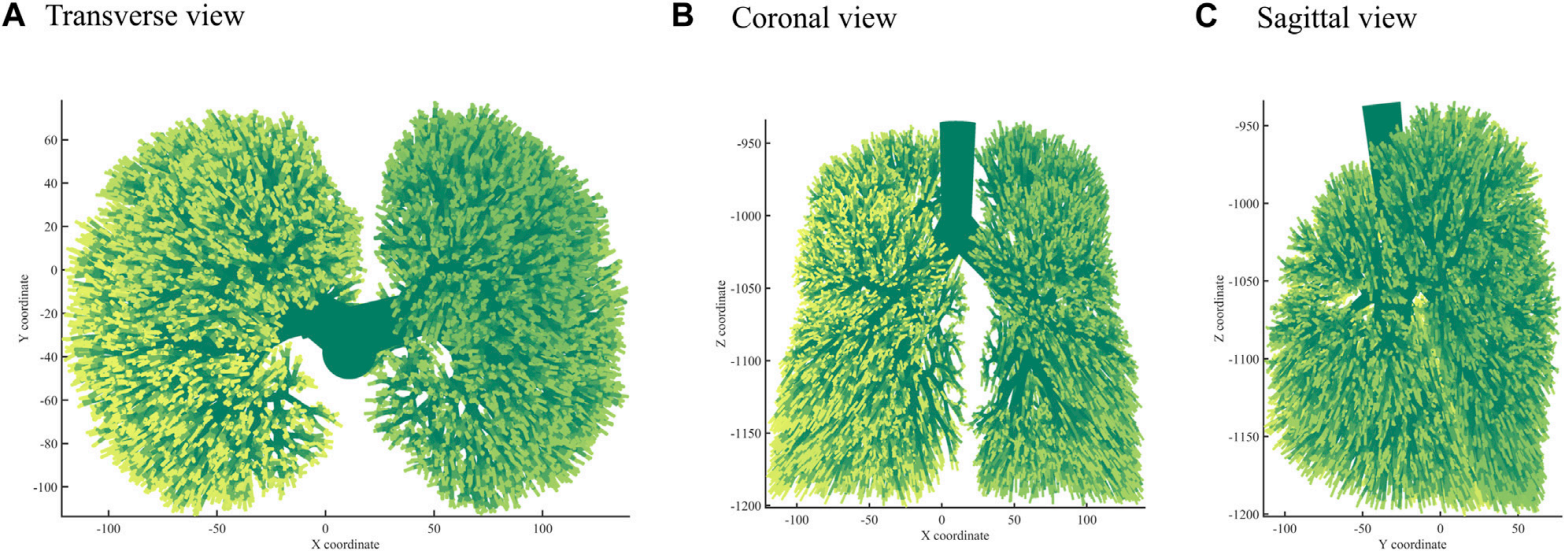
Three views: **(A)** Transverse view, **(B)** Coronal view, **(C)** Sagittal view of the 14th-generation bronchial tree model.

Next, we used the algorithm with selected coefficients to build a bronchial tree model with generations 10, 12, and 14 (Figure 3). Figure 4 shows the transverse, coronal, and sagittal view of the 14th-generation model. The 14th-generation model took approximately 8 h to calculate and 10 h to visualize with MATLAB R2023a using PowerEdge T430 with an Intel^®^ Xeon^®^ Gold 6128 CPU at 3.40 GHz and 3.39 GHz (two processors) and 64 GB RAM. The lung was filled evenly from generation to generation, from the 10th-generation model to the 14th-generation bronchial tree. Table 6 shows the performance index with the bronchial tree model of the 10th, 12th, and 14th generations. The failure branch rate had an obvious increase as each failure branch caused daughter branch failure too. A *t*-test showed that there was a significant increase when the generation went high (*p* < 0.05). The volume rate rises since more branches take more space. The coefficient of variation of terminal volumes $C_{v}$ drops a little bit, which means the small areas are now divided into more detailed small volumes, and they maintain a slight variation among those terminal volumes.

**TABLE 6:** Bronchial tree model of the 10th, 12th, and 14th generations with a performance index.

	$R_{fb}$	$V_{r}$	$C_{v}$
Grade 10	3.1%	32.59%	0.192
Grade 12	3.9%	42.59%	0.190
Grade 14	4.7%	57.02%	0.186

Table 7 shows the statistical size information of our constructed bronchial tree compared with the published data by Yeh and Schum (1980). The diameters of our bronchi are very close to their recorded data. The average length at different grades was close to the published data, too. Some generations were slightly longer, which makes more sense than the published data. The branches were supposed to drop in length from generation to generation. The turning angles were not that different to the published data, and they had the same kind of rise-drop pattern. We acquired some data from Kitaoka’s (2011) bronchial tree model. Their best performance model had a failure flow rate of 0.001, a volume ratio of 0.030, and a $C_{v}$ of acinar volume of 0.36. Our model had a failure branch rate of 0.031, a volume ratio of 0.3259, and a $C_{v}$ of terminal volumes of 0.192. To compare the performance, their model was better at controlling the flow rate and branch building but our model was better in terms of space division; they have a smaller volume ratio, which might be due to their smaller radius. Values for $C_{v}$ have been estimated to be 0.42 from 209 acini (Weibel et al., 1963), which also shows that our model has some merit in space division. In future studies, we will try to benchmark Kitaoka’s (2011) bronchial tree model and improve our successful branching rate. Overall, the performance looked good, and the statistical data matched the data posted by Yeh and Schum (1980).

**TABLE 7:** The size of our bronchial tree compared with the published data by Yeh and Schum (1980).

n	D (mm) ours	D (mm) published	L (mm) ours	L (mm) published	θ (deg.) ours	θ (deg.) published	V ( ${cm}^{3}$ ) ours	V ( ${cm}^{3}$ ) published
1	19.77	20.1	71.45	100	0	0	21.92	31.73
2	15.36	15.6	45.15	43.6	38	33	16.72	16.67
3	11.8	11.3	16.37	17.8	45	34	7.16	7.14
4	8.98	8.27	17.44	9.65	28	22	8.83	4.15
5	6.82	6.51	14.75	9.95	35	20	8.62	5.3
6	5.22	5.74	13.67	10.1	24	18	9.36	8.36
7	4.08	4.35	12.51	8.9	29	19	10.46	8.47
8	3.31	3.73	11.36	9.62	26	22	12.51	13.46
9	2.81	3.22	9.92	8.67	30	28	15.74	18.07
10	2.5	2.57	8.33	6.67	28	22	20.92	17.72

## 4 Discussion

Researchers have devised several methods for bronchial modeling over the past decades. Some have considered space occupation and some have considered flow rate distribution. Some methods are used within research groups and some are partially open for others to use. Our suggested algorithm is novel and comparatively more straightforward, with some controllable coefficients. It is easy to reiterate, and we will share the code upon request after the publication of this paper. Our bronchial tree model uses the starting points of a real human bronchial tree and grows in the real lung contour space, making it look more like a natural lung. In future studies, when we dig out more branching data from the extracted bronchial tree from specific CT images, we can make a patient-specific model, which can be more useful in clinical research. It is planned that patient-specific modeling will be used to solve the lung performance index, possibly lobularly, in the future.

## 5 Conclusion

We proposed three sets of algorithms to build the morphology model of the human bronchial tree and found that the third one performs better than the other two. From three performance indices, we have optimized the coefficients that control the modeling, and this could be used in future studies to make a patient-specific lung model, which can be more useful in the clinical research area. This model can be modified to build.stl files that can be used in the airflow simulation with finite element CFD study. Additionally, .stl files can be used in three-dimensional printing to make a realistic model with soft material. The lower airway model can later be modified to match different lung disease situations to help clinical research areas. Our group will also look for a friendlier way to let other researchers use our methods.

## Data Availability

The original contributions presented in the study are included in the article/Supplementary Material, further inquiries can be directed to the corresponding authors.
